# Guiding Microbial Crossroads: Syngas-Driven Valorisation of Anaerobic-Digestion Intermediates into Bio-Hydrogen and Volatile Fatty Acids

**DOI:** 10.3390/bioengineering12080816

**Published:** 2025-07-29

**Authors:** Alvaro dos Santos Neto, Mohammad J. Taherzadeh

**Affiliations:** Swedish Centre for Resource Recovery, University of Borås, 50190 Borås, Sweden; alvaro.dos_santos_neto@hb.se

**Keywords:** syngas fermentation, microbial consortia, hydrogen, volatile fatty acids

## Abstract

Anaerobic digestion (AD) has long been valued for producing a biogas–digestate pair, yet its profitability is tightening. Next-generation AD biorefineries now position syngas both as a supplementary feedstock and as a springboard to capture high-value intermediates, hydrogen (H_2_) and volatile fatty acids (VFA). This review dissects how complex natural consortia “decide” between hydrogenogenesis and acetogenesis when CO, H_2_, and CO_2_ co-exist in the feedstocks, bridging molecular mechanisms with process-scale levers. The map of the bioenergetic contest between the biological water–gas shift reaction and Wood–Ljungdahl pathways is discussed, revealing how electron flow, thermodynamic thresholds, and enzyme inhibition dictate microbial “decision”. Kinetic evidence from pure and mixed cultures is integrated with practical operating factors (gas composition and pressure, pH–temperature spectrum, culture media composition, hydraulic retention time, and cell density), which can bias consortia toward the desired product.

## 1. Introduction

Resource recovery from waste streams has emerged as a popular research topic as a way to give underutilised materials more value. Organic solid waste is one of the most applied resources for recovery, as most of this waste comes from animal, municipal, and agricultural wastes, which contain valuable nutrients (e.g., proteins, minerals, and sugars) that can be valorised [1]. From diverse types of organic solid waste, food waste has gained special attention and applicability as a source of high organic content that can be applied to produce biogas, hydrogen (H_2_), methane (CH_4_), chemicals (e.g., volatile fatty acids—VFAs), and others [2,3]. A commercial technology that can recover most of the potential of the food waste is anaerobic digestion (AD), where nutrients are digested by microorganisms in an anaerobic process.

With the versatility of the AD product range (e.g., VFA, biohydrogen, biogas, bio sludge), the concept of biorefinery for the AD process has been proposed to accelerate the achievement of a circular economy [4]. Stream products, such as digestate and biogas, have been commonly used, as their applications are the most straightforward for AD valorisation. Digestate, for instance, has been utilised as an agricultural fertiliser; if it is demonstrated to satisfy the necessary legislative requirements, it is allowed to be used on land [5]. Despite the fact that digestate contains important nutrients, like nitrogen, phosphorus, and potassium, the quality of digestate can vary based on process characteristics, like operational parameters and reactor configuration, which can result in unstable quality of digestate-derived fertilisers [5]. Similarly, the yield and composition of biogas can vary depending on the substrates used, operational parameters, and the type of digester [6]. Despite the high number of studies and the industrial application of AD, the interest in the main end products of AD (biogas and digestate) is reducing because of economic reasons, such as the increase in renewable energy sources (e.g., wind and solar generation) [6], and uncertainty regarding the application of digestate in agricultural fields [5]. Moreover, the economic feasibility of the process can be the main constraint for an AD biorefinery, even when the process is successful in producing renewable products, such as VFA and biogas [7]. Therefore, valorising AD products into higher-value goods, such as upgrading biogas to biomethane and integrating digestate with other technologies, is a promising strategy for developing a more appealing circular economy.

A promising technology that can integrate with the AD process is synthesis gas (syngas) fermentation [8]. Syngas fermentation is a process where waste gases, mainly carbon monoxide (CO), hydrogen (H_2_), and carbon dioxide (CO_2_), from thermochemical processes emissions (e.g., pyrolysis and gasification) or industrial emissions are coupled with a biological process employed by microorganisms in anaerobic conditions [9]. Syngas fermentation is a promising technology which can valorise the concept of resource recovery. The coupling of syngas fermentation with AD has been proposed for syngas biomethanation, where the gasification of the biomass can produce fermentable gas to be applied in a syngas fermentation process using biocatalysts, such as microbial consortia for biomethane production [10]. Moreover, there is a potential to couple syngas fermentation and AD to produce other biofuels and chemicals, such as H_2_ and VFA.

The utilisation of organic solid waste (e.g., microbial consortia) in a biological process employs the presence of a diverse microbial community, which can present several advantages compared to pure cultures, such as operation in non-sterile environments, higher adaptation capacity, and higher disturbance resiliency [10]. However, the diversity of microorganisms, which is one of the main advantages for their adaptability capacity, is also one of the main constraints, as diverse microbial pathways can be favoured with different operation parameters, directly impacting the yield of the desired end-product [11]. Therefore, the comprehension of how microorganisms interact and which metabolic pathways are selected by them is fundamental for the broader application of natural microbial consortia in processes, such as syngas fermentation [12]. The primary utility of this review is to bridge a crucial knowledge gap that currently limits the broader application of natural microbial consortia in industrial bioprocesses. We emphasise that a deeper understanding of microbial metabolic plasticity is essential for developing targeted strategies to guide bioprocesses, ultimately enabling the efficient and predictable synthesis of high-value products from syngas fermentation. Focusing on previous studies, which led to an understanding of microbial metabolism and the importance of operational parameters on the process, particularly for syngas fermentation, this work provides a novel comprehension of how microorganisms can be guided for specific end-products, especially H_2_ and VFA production.

This review comprehensively analyses the strategies employed by microorganisms to transition between metabolic pathways. A significant focus is placed on the H_2_ and VFA pathways, which are intermediate products of the (AD) process. The review begins with the description of H_2_ and VFA during AD and their potential applications. Furthermore, the potential usage of syngas fermentation with food waste to produce H_2_ and VFA is discussed. Therefore, the microbial “decision-making” is reviewed based on the current knowledge of the metabolic pathways and thermodynamic, kinetic, and process parameters that can influence the “decision-making”. Further current technological approaches and challenges for implementation are analysed, and future perspectives are presented.

## 2. Intermediate Products of Anaerobic Digestion: Volatile Fatty Acids and Hydrogen

The AD process is a complex and interdependent process where organic solid waste is digested into soluble organics, which are then converted to biogas and digestate [3]. For the achievement of this conversion, four biochemical reactions are needed, namely hydrolysis, acidogenesis, acetogenesis, and methanogenesis [13]. Acetyl-CoA is the precursor of acetate and the main intermediate to produce other VFAs, such as propionic, butyric, valeric, and caproic acid. Therefore, to simplify the explanation of the AD process, acetate will be used as the primary VFA produced in the subsequent reactions. In the first step, hydrolysis, the organic waste is transformed into soluble monomers and polymers by bacteria, as simplified in Equation (1) by the transformation of organic waste into a simple sugar (i.e., glucose) [14]. The later reaction, acidogenesis, is the process where VFAs (e.g., acetic acid—Equation (2), and propionic acid—Equation (3)) are produced from the degradation of monomers (e.g., sugars—glucose) from the hydrolysis reaction by acidogenic microorganisms [14]. In this phase, if H_2_ is not removed from methanogenic bacteria, the accumulated H_2_ can be used for other products, such as propionic acid (Equation (3)) [14].

(1) $$C_{6}H_{10}O_{4}+2H_{2}O\to C_{6}H_{12}O_{6}+2H_{2}$$

(2) $$C_{6}H_{12}O_{6}+2H_{2}O\to 2{CH}_{3}COOH+2CO_{2}+4H_{2}$$

(3) $$C_{6}H_{12}O_{6}+2H_{2}\to 2{CH}_{3}{CH}_{2}COOH+2H_{2}O$$

In the latter reaction, during acetogenesis (Equations (4) and (5)), the VFA produced during acidogenesis are converted into acetate (CH_3_COO^−^). Additionally, CO_2_ and H_2_ generated in the acidogenesis phase can be utilized by homoacetogens for acetate production (Equation (6)) [14].

(4) $${CH}_{3}{CH}_{2}OO^{-}+3H_{2}O+{CH}_{3}{COO}^{-}↔H^{+}{HCO}_{3}^{-}+3H_{2}$$

(5) $$C_{6}H_{12}O_{6}+2H_{2}O↔{2CH}_{3}{CH}_{2}OOH+2{CO}_{2}+4H_{2}$$

(6) $${CH}_{3}{CH}_{2}OH+2H_{2}O↔{CH}_{3}{COO}^{-}{+3H}_{2}+H^{+}$$

As AD is a syntrophic process, a symbiotic relationship exists within the microbial community, facilitating the process. For example, acetate produced during the acetogenesis reaction is responsible for the generation of electrons by syntrophic fatty acid bacteria, which generates H_2_ [15]. However, to keep a negative free energy, the H_2_ concentration should be kept low, making a symbiosis necessary between acetogenic and methanogenic bacteria, where methanogenic bacteria can use the H_2_ released and prevent toxic effects [15]. Moreover, acetate accumulation can also inhibit CH_4_ production, and, therefore, the syntrophic degradation of acetate is an important step in the AD process [13]. Therefore, several factors, such as the type of substrate and operating conditions, significantly influence the efficiency of CH_4_ production in the AD process, leading to a wide range of yields [14]. In particular, the use of municipal solid waste as substrate (e.g., food waste) results in highly variable CH_4_ outputs. Reported yields range from as low as 143 mL/g VS for cucumber waste to as high as 516 mL/g VS for fruit and vegetable waste [16]. This broad spectrum highlights the critical role of substrate composition in determining the overall CH_4_ potential production from waste streams.

As stated, AD can produce different product streams, such as CH_4_, VFAs, and H_2_. Therefore, the concept of biorefinery can be applied for VFAs and H_2_, as both are valuable intermediate products with a high market appeal [4]. VFAs, considered as building block chemicals, have numerous applications in various industries, such as the food, pharmaceutical, and chemical sectors [17]. H_2_ is considered a promising biofuel, but in addition to its application in renewable energy, it is also a valuable feedstock for the synthesis of chemicals [3]. As VFA and H_2_ are alternatives for the biogas production from the AD process, some research has focused on the production of these intermediates as the main products [18,19,20]. However, until now, to the best of the authors’ knowledge, the production of VFAs and H_2_ in a modified AD process has not been achieved on a commercial scale. Therefore, there is a potential to integrate an end-product from the AD process (i.e., digestate) into technologies with commercial production potential.

## 3. Syngas Fermentation: A Gateway for Intermediate Products Valorisation

**Figure 1:**
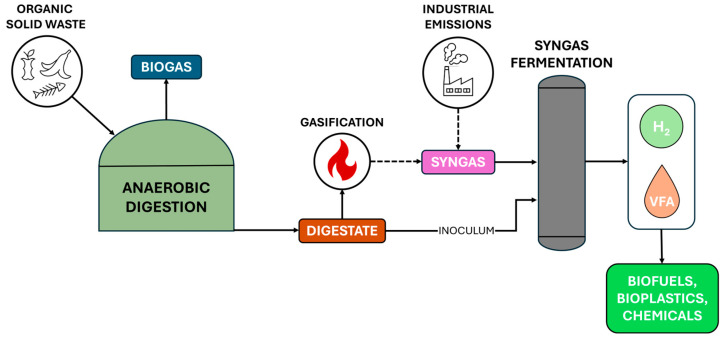
Coupling AD with syngas fermentation for AD intermediate products valorisation. Dashed arrows represent possible sources for syngas production.

A promising approach for valorising intermediate products from the AD process involves coupling AD with syngas fermentation. This integrated approach not only utilises syngas derived from the gasification of organic feedstocks but also leverages digestate as a potential inoculum source for syngas fermentation. Unlike AD, where the organic substrate serves as the primary carbon source for biogas/CH_4_ production, syngas fermentation relies on waste gases (mainly H_2_, CO, and CO_2_) as carbon and energy sources for carboxydotrophic microorganisms (i.e., microorganisms that can metabolise CO) [21]. Therefore, dual-stream valorisation, using both waste gases and AD-derived digestate, offers a unique opportunity to produce high-value intermediates, such as H_2_ and VFAs, which serve as precursors for biofuels, bioplastics, and chemicals (Figure 1).

As presented in a recently published review, syngas fermentation has achieved a high technological readiness level (TRL) for some companies producing ethanol, biopolymers, and protein ingredients, mainly using pure cultures for their process [9]. The utilisation of natural microbial consortia, such as those found in digestate, remains underexplored on an industrial scale, as the optimisation of the process can be challenging, not reaching the same levels as with pure cultures [22]. Despite these challenges, natural microbial consortia offer significant advantages, such as microbial resilience and adaptability to environmental factors (e.g., contaminants, heavy metals, different gas concentrations, and others) and the non-requirement for sterile conditions [23].

Initial studies demonstrated the feasibility of using natural microbial consortia for syngas fermentation, with successful conversions of CO into CH_4_ [24], acetate [25], and, more recently, H_2_ [26]. However, as in the AD, H_2_, and VFAs are intermediate products that can be further consumed by other microorganisms, such as hydrogenotrophic methanogens (Equation (7)) and homoacetogens (Equation (8)), in the case of H_2_ [27], and acetotrophic methanogens (Equation (9)) in the case of VFA conversion [28]. Therefore, to have H2 and VFA as end-products, these microbial reactions (Equations (7)–(9)) should be avoided or inhibited. Moreover, as H_2_ can be an intermediate product for VFA conversion (Equation (6)), the stability of the process can be challenging when H_2_ is the desired end-product. Investigating the mechanisms behind microbial “decision-making” in the conversion of H_2_ and VFAs is, therefore, essential for optimising product yields and enhancing the economic viability of this integrated bioprocess.

(7) $$4H_{2}+{CO}_{2}\to {CH}_{4}+2H_{2}O$$

(8) $$4H_{2}+2{CO}_{2}↔{CH}_{3}COOH+2H_{2}O$$

(9) $${CH}_{3}{COOH}^{-}+2H_{2}O\to 2{CH}_{4}+2{HCO}_{3}^{-}$$

## 4. Microbial “Decision-Making”

**Figure 2:**
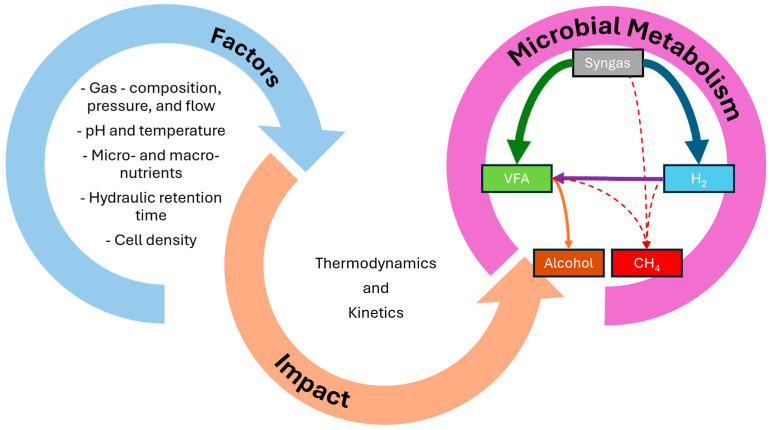
Impact of different factors on thermodynamics and kinetics which influence microbial metabolism. For the microbial metabolism part, thicker arrows represent the desired end-products, thinner arrows represent possible undesired routes, and dashed arrows represent inhibited pathways.

A natural anaerobic consortia, such as digestate, is a pool of different classes of microorganisms, which presents diverse substrate utilisation flux towards undesired end-products [29]. Therefore, it is fundamental to understand the mechanisms behind the metabolic flux towards specific end-products, such as H_2_ and VFA, as summarised in Figure 2. In this section, the metabolic pathways will be elucidated together with the thermodynamics and kinetics for the conversion into H_2_ and VFA. Moreover, as the environment has a fundamental impact on the metabolic flux direction, some aspects will be discussed to favour the flux direction into the specific end-product.

### 4.1. Overview of the Metabolic Pathways for Hydrogen and VFA Production

CO is well known as a toxic compound, and, in addition to its toxicity, some microorganisms can oxidise CO to be utilised in their autotrophic metabolism as an energy and carbon source [30]. These microorganisms, known as carboxydotrophs, are diverse, present in the Firmicutes and Proteobacteria phyla and even in the Archaea domain [31,32]. This ability to survive and grow in the presence of CO in anaerobic environments is determined by the presence of anaerobic carbon monoxide dehydrogenases (CODH) [33]. Through the identification of CODH genes in the microbial genome database, a study has shown that 6% of all bacteria and archaeal genomes sequenced encoded at least one CODH gene [33]. This finding suggests that a horizontal gene transfer may have occurred, sharing the capability of utilising CO among different microbial lineages by an evolutionary process, enhancing the adaptability of these microorganisms to survive in the presence of CO [33]. Furthermore, these microorganisms utilise CO as a fuel for numerous metabolisms, such as hydrogenogenesis, or the so-called biological water–gas shift (WGS) reaction, and acetogenesis, or the so-called Wood–Ljungdahl pathway, for the conversion of CO into H_2_ and VFA, respectively [31].

#### 4.1.1. Biological Water–Gas Shift Reaction

**Figure 3:**
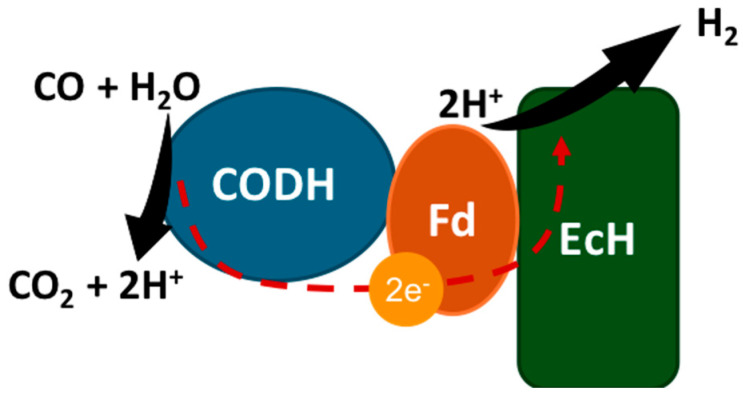
Biological water–gas shift reaction representing CO oxidation for H_2_ production. CODH: carbon monoxide dehydrogenase; Fd: ferredoxin; EcH: energy-converting hydrogenase.

Hydrogenogenic carboxydotrophs are the microorganisms that perform the biological WGS reaction for the conversion of CO into H_2_ by Equation (10). This conversion is a mechanism to conserve metabolic energy through the formation of H_2_ via the CO oxidation and proton reduction by the CODH, an electron transfer protein, and an energy-converting hydrogenase (EcH), as illustrated in Figure 3 [34,35]. The initial step is the oxidation of CO by the CODH, which releases electrons that are transferred to a “ferrodoxin-like” electron carrier, which is further oxidised. Consequently, a proton reduction occurs via the EcH, which releases H_2_ and translocates protons or sodium ions through a membrane to generate a chemiosmotic ion gradient that leads to ATP synthesis by an ATP-synthase [35]. Therefore, in this pathway, CO is not used as a carbon source, but, instead, it is used for energy, as metabolic energy is conserved during this reaction (Equation (10)).

(10) $$CO+H_{2}O↔{CO}_{2}+H_{2}$$

#### 4.1.2. Wood–Ljungdahl Pathway

The Wood–Ljungdahl pathway, also so-called the reductive acetyl-CoA pathway, is the pathway where the reduction of H_2_, CO_2_, and CO occurs by acetogenic bacteria [36]. This pathway consists of two branches, the methyl and carbonyl branches, where the intermediate step, acetyl-CoA, is formed [37]. Besides CODH, numerous enzymes are responsible for several carbon reduction reactions that occur to form acetyl-CoA, which is the precursor of several products, such as acetate and ethanol [9]. Besides the ability to reduce H_2_, CO_2_, and CO, acetogens also reduce other compounds, such as sugars, formate, and methanol [9,38]. As the aim of this review is to elucidate microbial “decision-making” in natural microbial consortia under a syngas fermentation process, the explanation of the Wood–Ljungdahl pathway focuses on CO as the main substrate.

**Figure 4:**
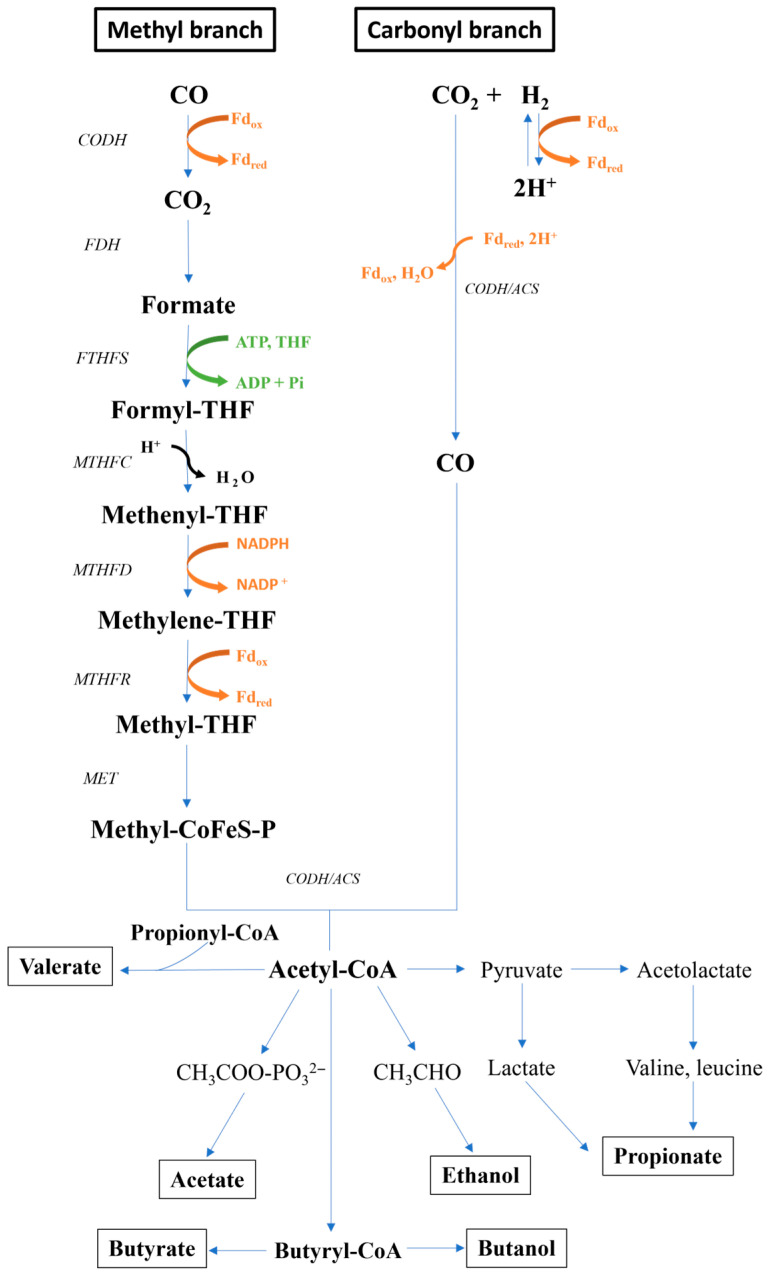
Wood–Ljungdahl pathway with VFAs (i.e., acetate, butyrate, propionate, and valerate) and alcohols (i.e., ethanol and butanol) as end-products. CODH: carbon monoxide dehydrogenases; FDH: formate dehydrogenase; FTHFS: formyl-THF synthase; MTHFC: methenyl-THF cyclohydrolase; MTHFD: methylene-THF dehydrogenase; MTHFR: methylene-THF reductase; MET: trans-methylase.

Firstly, in a carbon fixation process, CODH oxidises one molecule of CO into CO_2_ and then the other molecule proceeds into the carbonyl branch directly [36]. CO_2_ enters the methyl branch, where it undergoes a series of reductions leading to the formation of acetyl-CoA, as illustrated in Figure 4 [36,39]. Initially, CO_2_ is reduced to formate by the formate dehydrogenase (FDH) enzyme. Then, formate is activated by tetrahydrofolate (THF) to form formyl-THF, which is synthesised by formyl-THF synthase (FTHFS), in a consuming adenosine triphosphate (ATP) step. Further, formyl-THF is reduced to methyl-THF by different THF-dependent enzymes, such as methenyl-THF cyclohydrolase (MTHFC), yielding methenyl-THF, methylene-THF dehydrogenase (MTHFD), which reduces methenyl-THF to methylene-THF, and lastly, methylene-THF reductase (MTHFR) to form methyl-THF. At the end of the methyl synthesis, the methyl group is transferred to a corrinoid iron–sulphur protein (CoFeS-P), where the reduction of the methyl group is carried out by reduced ferredoxin. To obtain a reduced ferredoxin, organisms have different possibilities, although when CO is the substrate, CO oxidation to CO_2_ yields directly reduced ferredoxin [40]. Then, CoFeS-P is methylated by trans-methylase (MET), forming methyl-CoFeSP, finishing the reductions in the methyl branch. To combine the carbonyl and methyl branches, a complex from CODH and acetyl-CoA synthase is formed (CODH/ACS). By combining the two branches with coenzyme A, CODH/ACS yields acetyl-CoA, which is the intermediate step to produce several products (e.g., VFA, ethanol, butanol, and others). For acetate (two-carbon VFA-C2) production, a substrate-level phosphorylation (SLP) mechanism is coupled with the reaction, generating one molecule of ATP per mole of acetate produced [38]. A zero net ATP production is yielded during acetate production, as one mole of ATP is used in the activation of formate to formyl-THF. Therefore, microorganisms rely on indirect chemiosmotic mechanisms, such as the membrane-associated electron transport chain and proton motive force, which have been presumed to act in this pathway for the generation of ATP, contributing to the energy conservation process [41]. For ethanol production, acetyl-CoA is reduced to acetaldehyde by an aldehyde/alcohol dehydrogenase (ADHE). However, based on the sequenced acetogens, which contain genes encoding aldehyde–ferredoxin oxidoreductase (AOR), it is assumed that ethanol production is preferentially formed by acetate reduction to acetaldehyde, by AOR, to be further converted to ethanol by ADHE, contributing to energy conservation [36]. By a chain elongation process, other VFAs (i.e., butyrate—C4 and caproate—C6) can be formed by a reverse β oxidation reaction, where two-carbon units (derived from acetyl-CoA) act as a primer to several chemical reactions (i.e., C2 to C4 to C6) catalysed by different enzymes [42]. Another alcohol, butanol, is a product of the reverse β oxidation reaction, where butyryl-CoA, instead of being converted to butyrate, can be converted to butyraldehyde and then to butanol by ADHE [36,43]. Other VFAs, such as propionate and valerate, come from the conversion of other products from the Wood–Ljungdahl pathway. The conversion to propionate can occur via the amino acid catabolic pathway, acrylate pathway, or biosynthetic pathways [44]. For valerate, the production comes from the association of propionyl-CoA and acetyl-CoA in the Wood–Werkman cycle, which is predominantly found in Propionibacterium, in a process that elongates propionate to valerate [44,45].

### 4.2. Thermodynamics

Microorganisms must use the energy released from redox reactions derived from their environment to survive. Hydrogenogenic and acetogenic microorganisms catalyse redox reactions where electrons are transferred from a donor to an acceptor species, making the donor oxidised and the acceptor reduced [46]. Then, part of the energy liberated (Gibbs free energy—∆G) is conserved by the translocation of a proton outside of the cell’s membrane to synthesise ATP [46]. Because of the competition between metabolisms, microorganisms are committed to maximising their thermodynamic efficiency in ATP synthesis (ATP/∆G) [47]. Using the metaphor example by Wang, Sun, and Zhu [48], a natural microbial consortia is a complex social economy, where competitive relationships occur between different metabolic pathways because of their specific thermodynamics, which are controlled by their energy yields, following the highest to the lowest energy yields.

CO is a low-potential energy source, as described by the redox potential (E^o’^) on the reversible oxidation of CO to CO_2_ (E^o’^ = − 520 mV) [47]. Even though some microorganisms can rely solely on CO or on a combination of other energy sources for their growth [49], in anaerobic conditions, the biological WGS reaction (Equation (10)) has a standard ∆G change per mol of CO of −20 kJ/mol, which makes this reaction thermodynamically favourable, even presenting a relatively small ∆G [49,50]. The biological WGS reaction is not an exception; many other anaerobic conversions proceed close to thermodynamic equilibrium (∆G = 0), which makes essential the syntropy between microorganisms to survive at the thermodynamic edge of life [51]. However, in processes applying a natural microbial consortium, the syntrophic relation between microorganisms could not be favourable when a specific metabolic route is aimed for.

Anaerobic CO oxidation always relies on the biological WGS reaction, which is used as a route to increase the energy yield in other metabolic pathways [49]. In a syngas fermentation, it would be more efficient to obtain electrons from H_2_ instead of CO, as the carbon source could be utilised for the conversion of carbon-containing products (e.g., acetic acid), but the concentration of H_2_ in a syngas composition may not be enough to provide all the electrons needed [52]. Based on the study of Hu, Bowen, and Lewis [52], the electron production from CO is always thermodynamically favourable, independent of the process conditions. This favourable condition for electron production from CO directly competes with the production of acetyl-CoA, the intermediate product for acetic acid production, which reduces the conversion efficiency into cell mass and products. For instance, for acetic acid production, there is an advantage when the conversion is based on CO (Equations (11)–(14)), as with the decrease in the CO content, there is a decrease in the ∆G, favouring the reaction, and in the absence of CO, the ∆G for acetic acid production is the highest one (Equation (15)) [43,53].

(11) $$\begin{array}{ll} 4CO+H_{2}O\to {CH}_{3}COOH+2{CO}_{2} & \;\Delta G=-156.6kJ/mol \end{array}$$

(12) $$\begin{array}{cc} \;3CO+{H_{2}+H}_{2}O\to {CH}_{3}COOH+{CO}_{2} & \Delta G=-134.5kJ/mol \end{array}$$

(13) $$\begin{array}{cc} 2CO+2H_{2}\to {CH}_{3}COOH & \;\Delta G=-114.5kJ/mol \end{array}$$

(14) $$\begin{array}{cc} CO+3{H_{2}+CO}_{2}\to {CH}_{3}COOH+H_{2}O & \Delta G=-94.4kJ/mol \end{array}$$

(15) $$\begin{array}{cc} 4H_{2}+2{CO}_{2}\to {CH}_{3}COOH+2H_{2}O & \;\Delta G=-75.4kJ/mol \end{array}$$

The understanding of what drives the redox potential is essential to accurately predict microbial metabolism. The environment, in addition to providing the habitat and the nutrients for the microorganisms, also provides the energy sources that can shift not only the microbial community but also their metabolic reactions [46]. Therefore, the environmental impact on H_2_ and VFA production will be discussed in Section 4.4.

### 4.3. Kinetics

Understanding microbial kinetics is key to clarifying the process, but accurate tools are needed to measure substrate levels, mass transfer, and uptake rates in syngas fermentation [54]. Moreover, the kinetics studies available on CO conversion into acetic acid are based on pure cultures [54,55,56]. The same is true for H_2_ conversion, where a single study has evaluated the kinetics of *Carboxydothermus hydrogenoformans* [57]. Most of the kinetic studies available using microbial consortia are based on CH_4_ production [58,59,60] or ethanol [61]. Therefore, assumptions for the kinetics of the conversion of syngas into H_2_ or VFAs will be based on the studies discussed above.

Based on pure culture studies, CO has a significant inhibitory effect on the process. However, even when using pure cultures, the sensitivity to CO from different species can be lower or higher, which is an advantageous aspect when mixed cultures are applied. Even in Clostridium species, there is a vast difference in CO tolerance for acetate production, as summarised in Table 1. *Clostridium aceticum* is highly sensitive to CO, with an optimal CO partial pressure of only 5.4 mbar [55], in contrast, *Clostridium autoethanogenum*, which has performed in a partial pressure 100× higher (~600 mbar) [54]. In another study investigating the kinetics of syngas conversion to acetate by *Acetobacterium wieringae* and Clostridium species (*C. autoethanogenum* and *C. carboxidivorans*), the authors could reveal distinct stoichiometry patterns between the species evaluated [56]. *C. carboxidivorans* exhibited a fixed stoichiometry, where 4.0 mol of CO was consumed to produce 1.5–2.0 mol of CO_2_ per mol of acetate. In contrast, *C. autoethanogenum* consumed 1.8–3.0 mol of CO, 2.0–6.0 mol of H_2_, and 0.2–1.2 mol of CO_2_ to produce acetate. Finally, *Acetobacterium wieringae* displayed a higher CO demand, at lower CO concentrations, to produce acetate, also favouring CO_2_ production. Similar to acetate production, H_2_ conversion by *C. hydrogenoformans* also presents an inhibitory effect based on the CO concentration [57]. An optimum CO concentration was registered around 1000 mbar; beyond that, the CO consumption presented a sharp decrease.

To accurately describe the process, different kinetic models can be used. Mayer, et al. [55] have used a substrate inhibition model (Andrews model) to identify the CO inhibition kinetics and the acetate formation rates of *C. aceticum*. To evaluate the optimum CO concentration for H_2_ conversion, another inhibition model (the Han and Levenspiel model) was used by Zhao, et al. [57]. Another study has used a dynamic kinetic model incorporating different aspects, such as the gas–liquid mass transfer rate, CO uptake rate, and three different conversion pathways (acid/alcohol production associated with cell growth, alcohol production by acid conversion, and direct alcohol production not associated with cell growth) [63]. Regarding studies using microbial consortia, one main disadvantage as compared with pure cultures is that most of the models used treat the microbial consortium as a single individual or a few group interactions, which makes some pathways unseen. Simple and common approaches can be used for microbial consortia, such as the Monod model applied by Ako, et al. [59] and the Gompertz model applied by Pan, et al. [60]. Using a thermodynamic potential factor, a study has used the ΔG of different metabolic pathways as a tool in kinetic models to evaluate if a metabolic pathway is favourable enough to proceed, which, therefore, makes the reaction thermodynamically controlled instead of kinetically controlled [61]. In a further study, the same author modelled a process incorporating several microbial interactions, such as cross-feeding, competition, syntrophic interactions, and different microbial pathways (e.g., acetogenesis, methanogenesis, hydrogenogenesis) to make the kinetics consistent with the thermodynamic feasibility [58].

Based on these studies, the microbial “decision” in a consortium for H_2_ and VFA production kinetics in a syngas fermentation process is complex. Both products, H_2_ and VFA, are highly dependent on microbial composition, the interactions, and the process parameters that can favour their production or make unfavourable conditions for their consumers. Therefore, environmental factors will be discussed in the following Section 4.4.

### 4.4. Environmental Factors and Their Impact

The presence of a toxic gas, such as CO, creates a hostile environment; nevertheless, many microorganisms are capable of survival. The ability of CO oxidation by microorganisms comes from the early atmospheric conditions, when oxygen was not available, around 4 billion years ago [64]. Nowadays, CO is not heavily concentrated in the atmosphere (a few ppm on average); nonetheless, these microorganisms have survived in diverse ecological niches, such as terrestrial, aquatic, and even anaerobic sludge [65,66]. However, anthropogenic activities, such as traffic, combustion of solid waste from landfills and agricultural waste, and industrial processes (e.g., emissions from steel mills), are currently the main CO emitters [67]. Biotechnological approaches, like syngas fermentation, can reduce emissions while producing renewable resources, such as H_2_ and VFA, supporting the development of a circular bioeconomy. The production of these specific products is strongly influenced by environmental conditions, which shape microbial metabolism. The following sections will explore how process parameters impact the environmental conditions. The data discussed in the next sections regarding the production of H_2_ and VFA are based on processes that have inhibited or mitigated the production of CH_4_, or on assumptions based on the intermediate production of H_2_ and VFA in CH_4_ production processes. Therefore, the inhibition of CH_4_ will not be discussed in this review, as different inhibition methods, such as the use of target inhibitors (e.g., 2-bromoethanesulfonic sodium–BES) [26] and thermal treatments have been effectively applied for methanogen suppression [27].

#### 4.4.1. Gas Composition

Gas is one of the most important environmental factors in a syngas fermentation process, as it has a significant impact on the gas–liquid mass transfer because of the gas components’ solubilities [68]. Gas can impact not only the gas–liquid mass transfer but also the microbial community and metabolism [23]. Therefore, insights regarding the impact of gas on the microbial decision for H_2_ or VFA conversion can be assumed.

As part of the metabolic process of many anaerobic microorganisms, the conversion of H_2_ into protons and electrons and the reverse, H_2_ production, is a vital step catalysed by specialised enzymes called hydrogenases [69]. Hydrogenases are classified according to their metal content in the active site into [Fe], [Ni-Fe], or [Fe-Fe] hydrogenases [69]. CO is a well-known inhibitor of hydrogenases, with the inhibition mechanism varying depending on the type of hydrogenase [69]. For [Ni-Fe] hydrogenase, a study has elucidated the role of its gas channels during CO inhibition [70]. CO and oxygen have shown similar diffusion rates through the protein channels, but CO inhibition was faster, showing how quickly CO can react with the active site. The gas channels represent a possible solution for hydrogenases to slow down the CO entry and, therefore, decrease the rate of CO inhibition. However, it can also impact H_2_ diffusion, decreasing the enzymes’ overall efficiency. In another study, the inhibition mechanism of hydrogenases was evaluated in the presence of H_2_, which is also another potential inhibitor of H_2_ production [71]. In [Ni-Fe] hydrogenases, a phenomenon known as product inhibition can occur, as the H_2_ production can be inhibited by the H_2_ produced from protons and electrons (product H_2_). By the observation of a series of channel mutants, a study demonstrated that H_2_ diffusion rates can present a decrease towards the active site, which causes H_2_ inhibition by the H_2_ crowding in the gas channel. In the same study, the effect of H_2_ concentration on H_2_ production by [Fe-Fe] hydrogenases was evaluated, showing that H_2_ concentration has a direct impact on the hydrogenase inhibition, but with a smaller effect than for [Ni-Fe] hydrogenases, as the inhibition constant is larger for [Fe-Fe] hydrogenases (about 50 times). This finding means that [Fe-Fe] hydrogenases are efficient catalysts of H_2_ production. Regarding the inhibition of [Fe-Fe] hydrogenases by CO, it was found that CO inhibits both H_2_ oxidation and H_2_ production, as CO strongly binds to the active site, preventing the enzyme from catalysing the reactions [72].

In addition to the impact of the gas composition on hydrogenases, other studies have elucidated its impact on microbial metabolism. In the study of Grimalt-Alemany, et al. [58], it was suggested by the kinetics parameters and thermodynamics of a microbial consortia that when the CO concentration decreases and the H_2_ concentration increases, this condition could favour the dominance of homoacetogenic microorganisms (Equation (6)). In the same study was proposed that hydrogenogenesis could be thermodynamically favoured by modulating the pressure of CO_2_, as at a decreasing CO_2_ pressure, the thermodynamics would be higher for hydrogenogenesis, increasing the H_2_ production. In a study applying a dilution of syngas (35:30:25:10%*v*/*v* H_2_–CO–CO_2_–CH_4_), it was noticed that with higher dilutions (25% and 50% of the original concentration), a lower acetate production was obtained in association with the lower production of biomass. In contrast, at higher concentrations (75% and 100%), a high acetate production was obtained, but with a decrease in microbial activity due to the pH drop [73]. Another study evaluated the impact of the addition of H_2_ and/or CO_2_ in the CO fermentation process on the microbial metabolism of pure and CO-enriched mixed culture [74]. The addition of H_2_ and CO_2_ affected the metabolism of carboxydotrophs, especially in pure cultures, which have shown an inhibition in CO oxidation in the presence of CO_2_, while the mixed culture could maintain de CO consumption rates and consume H_2_ and CO_2_ along with CO, shifting the production from acetate to ethanol. Using modelling and simulation tools, a study has shown that CO conversion is affected by gas flow rates, which are inversely related to the empty bed residence time (EBRT) in a trickle bed reactor. Varying the EBRT on the simulations showed that a higher EBRT, or a lower gas flow rate, make it possible to achieve higher CO conversion efficiency towards H_2_ [75].

#### 4.4.2. pH and Temperature

In addition to the gas composition and concentration, the pH and temperature of the process can affect the product pathway, as both have a direct impact on the thermodynamics of product reactions [43]. The pH value has a direct impact on the process, as it directly affects the proton concentration in the culture media [76]. A lower pH leads to a higher proton concentration, which can reduce protons to H_2_ in a more thermodynamically favourable way. When pH changes in the environment, microorganisms regulate their intracellular pH to maintain their enzyme activities and metabolic functions. For example, when acetic acid is released into the medium, its undissociated form causes a decrease in the internal pH by the diffusion of the protons inside the cells, as in their undissociated form they are more permeable to cell membranes [77]. In this condition, with an accumulation of VFA and a decrease in the pH, a shift in the metabolism of the microorganisms can happen. Instead of VFA production, which is favourable at neutral pH values, a decrease in pH will favour solventogenesis, and, therefore, the production of ethanol [78]. Moreover, pH values can shift the microbial metabolism to different pathways and products [9], optimum pH values can also vary between species or different microbial consortia (Table 2) and can also be a potential inhibiting factor, depending on the optimum pH values between different groups of microorganisms [61].

As presented in Table 2, the optimum temperature values can also vary between species or sources of microbial consortia. As described by Harahap and Ahring [77], several studies have found different optimum temperature ranges for specific species of acetogens, showing a decrease in production with an increase in temperature, or also the opposite, highlighting the importance of temperature optimisation for the process. In the case of H_2_ production, a favourable aspect regarding the temperature shifting is demonstrated by hydrogenases, which can present a good stability in different temperature ranges, functioning even at elevated temperatures [69]. Moreover, temperature also impacts gas solubility, as when increasing the temperature, the gas solubility decreases, lowering the availability of gases in the liquid phase and impacting cell growth and production [77]. A study using dynamic mass transfer modelling has demonstrated that the negative effect on the decreased gas solubility at thermophilic temperature (60 °C) is suppressed by the positive effect on the enhanced mass transfer coefficient ($k_{L}a$), showing a positive effect on CH_4_ production [88]. Therefore, the impact of temperature on the $k_{L}a$ to produce H_2_ and VFA should also be evaluated for temperature optimisation.

#### 4.4.3. Culture Media Components

In syngas fermentation, CO and CO_2_ serve as the main carbon sources, while macro- and micronutrients support the conversion of H_2_ or VFAs. Nitrogen is an essential macronutrient, being a fundamental constituent of microbial amino acids and proteins, with a vital role in microbial growth [89]. In a dark fermentation process to produce H_2_, the impact of carbon to nitrogen (C/N) ratio was evaluated in a hydrogenogenic mix culture with the supplementation of ammonium chloride as a nitrogen source, where high C/N ratios (≈135) present a lower H_2_ production compared with a decreased C/N ratio (≈41) using a tequila vinasse wastewater with a high organic load as substrate [90]. For VFA production, a co-fermentation study with syngas and carbohydrate-rich synthetic wastewater (glucose as the model compound) could enhance the conversion, favouring acetogens. However, with a protein-rich synthetic wastewater (bovine serum albumin as the model compound), the conversion was inhibited due to a high concentration of ammonium (>900 mg/L) [91]. Therefore, high concentrations of nitrogen can impact both H_2_ and VFA production. In a study using different biochars in the fermentation media, the biochar which contained the highest phosphorus content, poultry litter, was correlated to an increase in the acetic acid concentration, which was further converted to ethanol by the solventogenesis phase from *C. ragsdalei* [92]. Another study has shown that phosphorus limitation directly impacted the cell growth of *C. carboxidivorans* without impacting ethanol production [93]. As ethanol is a product of the Wood–Ljungdahl pathway, VFA or acetic acid production could also be impacted by phosphorus limitation. Beyond macronutrients, like carbon, nitrogen, and phosphorus, complex media components, such as micronutrients (e.g., mineral salts and vitamins), can significantly influence the final product, as summarised in Table 3.

Ni and Fe are important trace metals, especially for the activity of hydrogenases. In an anaerobic fermentation with glucose as the carbon source for H_2_ production, a 100x increase in Fe^2+^ concentration could enhance H_2_ production by 71%, which was similar to a 50x higher concentration of Ni^2+^ [98]. For VFAs, Ni and Fe can impact their production as more H_2_ can be generated and, therefore, converted to VFAs by homoacetogens. For example, Bayar, Veiga, and Kennes [99] have demonstrated that the addition of Fe^2+^, specifically zero-valent iron (ZVI), could enhance the generation of H_2_ by the interaction of ZVI with water, which was, therefore, an electron donor for CO_2_ reduction by *C. aceticum* and *C. carboxidivorans* for acetic acid production. In contrast, a study observed that an increase in acetate production was registered when Fe^2+^ was absent [94]. However, in the case of Ni^2+^, the metal was essential for the growth of *C. ragsdalei*, and a 10x increase in Ni^2+^ concentration could enhance acetate production by 24%. Cobalt (Co) is an essential trace metal as it is a constituent of a key enzyme (CoFeS-P) in the Wood–Ljungdahl pathway, which is fundamental for acetyl-CoA synthesis [95]. Although it is an essential trace metal, an acidogenic fermentation study on propionic acid production showed that increasing Co^2+^ concentration led to reduced VFA yields and even inhibited propionic acid formation [100]. Another trace metal that indirectly influences VFA production is molybdenum (Mo). At relatively low concentrations of Mo, cell growth and alcohol synthesis are promoted, as in acetogens, Mo and tungsten are cofactors of FDHs, which are responsible for catalysing CO_2_ to formate in the Wood–Ljungdahl pathway (Figure 4) [96]. Other trace metals, such as copper (Cu), zinc (Zn), and manganese (Mn), are important for different enzymes. As summarised by Chandolias, et al. [97], Mn is important for the stabilisation of the methyltransferase, Cu and Zn can affect the hydrogenase in the methyltransferase, and Zn can affect the activity of FDH. Therefore, in the study of Chandolias, et al. [97], it was observed that Cu, Zn, and Mn at low concentrations (i.e., 0.04–0.1, 0.25–0.67, and 1.06–2.8 mg/L, respectively) could enhance H_2_ production at all pH levels evaluated (i.e., 5, 6, and 7). A further increase in these metal concentrations (i.e., Cu [0.625], Zn [3.75], and Mn [17.5] mg/L) presented an inhibitory effect on H_2_ production, except for pH 5, and at higher concentrations, H_2_ production was inhibited in all evaluated pH values. Riboflavin, or vitamin B2, is a biogenic biocatalyst which has been reported to act as an extracellular redox mediator [82]. A study on fermentative VFA production from waste sludge demonstrated that riboflavin supplementation at 25 °C and 35 °C enhanced VFA yields, indicating a stimulatory effect under moderate temperature conditions [82]. Moreover, other sources of nutrients, such as yeast extract, can impact the end-product conversion in syngas fermentation. In a study using *C. autoethanogenum* in a syngas fermentation process, yeast extract was correlated to an increase of 74% in the acetic acid production with a 10x increase in yeast concentration [101].

#### 4.4.4. Hydraulic Retention Time, Gas Flow Rate, and Cell Density

Hydraulic retention time (HRT) or dilution rate is a fundamental parameter in continuous bioprocesses as it dictates the washout rate of microorganisms and the average length of soluble compounds in the system. Knowing the kinetics of the desired group of microorganisms for the specific product is fundamental, as it can favour different microorganisms based on their growth rates and the change of the HRT. A study using anaerobic sludge in a hollow-fibre membrane biofilm reactor has evaluated the impact of HRT on acetate production [102]. Acetate concentration decreased from 19.3 g/L to 10.5 g/L when HRT was changed from 2.5 days to 1 day, although the production rate was 30% higher at a shorter HRT. However, it was observed that the shortest HRT (i.e., 0.5 day) could not keep steady, probably due to the high washing rate of microorganisms. In a dark fermentation process for H_2_ production from the organic fraction of municipal solid wastes, three HRTs were evaluated (i.e., 4, 5, and 6 days), showing the highest H_2_ production with an HRT of 5 days, presenting a 28% higher H_2_ production than the HRT of 4 days. The shortest HRT (i.e., 4 days) presented the highest VFA concentration, and the longest HRT (i.e., 6 days) favoured methanogens, showing CH_4_ production [103]. Therefore, to produce H_2_ or VFA, shorter HRTs are favourable. A competitive dynamic between their metabolic pathways can happen, which makes it essential to optimise HRT for the specific product.

Another important parameter in continuous processes is the gas flow rate, as it directly impacts the conversion efficiency of the gases into the specific products. As described in Section 4.3. the CO oxidation always relies on the biological WGS reaction. Therefore, studies showing CO conversion for different products can be correlated with H_2_ production, as it is an intermediate product for other reactions. In the study of Asimakopoulos, et al. [104], the authors demonstrated a complete conversion of CO at lower gas flow rates for biomethane production. Even with a low electron yield to VFA production (<0.7%), an interesting result was observed regarding the gas flow rates evaluated, where, at low rates, acetic acid was the main byproduct in the liquid; in contrast, at higher gas flow rates, propionic acid was dominant; thus, the authors assumed that scavengers of dead cells oxidised complex organic compounds to produce propionic acid. The impact of different gas flow rates was also evaluated in a monolith-based biofilm reactor in syngas fermentation [105]. An increase in the gas flow rate from 50 to 300 mL/min increased CO and H_2_ consumption, as well as the acetic acid and ethanol concentration. However, a further increase to 500 mL/min decreased the gas consumption, and no further increase in acetic acid and ethanol concentration was observed, showing that the gas supply exceeded the cell’s conversion capacity, presenting a kinetic growth limitation.

Cell density is another important parameter that can be controlled during the process, showing different optimum ranges as described in Table 4. Even in a batch process, the impact of the cell density can be evaluated. Using a microbial consortium to produce H_2_, a study evaluated different volatile suspended solids (VSSs) concentrations and their impact on H_2_ production and CO conversion in batch modes [27]. The highest H_2_ productions were observed at 20 and 30 g/L of VSSs, and minimal H_2_ production and CO conversion were observed at lower concentrations. At higher concentrations, high CO conversion efficiency was observed, but H_2_ production was lower than with the two best concentrations, as the heat treatment applied was inefficient for suppressing the methanogens’ activity at high concentrations, leading to the CO conversion to CH_4_. Another study, using a pure culture (i.e., *Clostridium ljungdahlii*), evaluated the impact of cell density and the influence of gas flow on product formation in a continuous syngas fermentation process [106]. The use of a cell retention system increased the cell density by 160%, which led to an increase in ethanol production and a decrease in acetic acid formation. The gas flow also had an impact on product formation; with an initial 31% increase in the gas flow, a 19% increase in cell density was observed, with a slight increase in the acetic acid to ethanol product ratio. A further 17% increase in the gas flow resulted in a 34% decrease in the cell density and a product shift towards acetic acid, which was correlated with an increase in CO-induced inhibition of hydrogenase.

## 5. Current Technologies and Challenges for Implementation

Syngas fermentation has attracted the attention of the scientific community, as well as the industry, as a potential technology to convert waste gases and low-cost organic substrates into value-added chemicals and biofuels [9,39]. As summarised in a recent review [9], several companies are applying syngas fermentation at different technology readiness levels (TRLs) for diverse end-products. However, to the best of the authors’ knowledge, no commercial facility is yet targeting H_2_ or VFAs as final products. To illustrate the potential of syngas fermentation for these compounds, some research initiatives are briefly discussed.

For instance, the SynoProtein project applies syngas fermentation to convert syngas into H_2_ and acetic acid, which are subsequently used as substrates for microbial protein production [107]. Similarly, the CO2SMOS project focuses on syngas fermentation to achieve a biotransformation of CO_2_ into acetate and 2,3-butanediol, which are then used in a second fermentation as the carbon source to produce biopolymers, such as polyhydroxyalkanoate (PHA) and polyhydroxybutyrate (PHB) [108]. These examples demonstrate the flexibility of syngas fermentation and the broad applicability of H_2_ and VFA in different fields, highlighting their market appeal.

While syngas fermentation is recognised as a promising technology for the decarbonization of waste streams and a valuable process for the production of renewable chemicals and biofuels, it continues to encounter numerous obstacles that impede its widespread industrial application. One of the main technical limitations is the low solubility of syngas components, such as CO and H_2_, which restricts mass transfer and microbial uptake [77]. To address this, various strategies have been explored, including the use of pressurised systems [80] and advanced reactor configurations, such as a hollow-fibre membrane biofilm reactor [87] and trickle bed reactor [88]. However, there is a certain resistance from industries regarding the choice of their reactor type, which can limit the commercialisation of syngas fermentation processes because of the high capital costs and small economic margins from the low-value product spectrum [109].

Another significant challenge lies in the choice of microbial systems. While current industrial processes rely on pure cultures for their high specificity and yield, these systems require sterile conditions and are sensitive to environmental fluctuations. In contrast, natural microbial consortia, such as those found in digestate, offer advantages, like resilience to contaminants and reduced operational costs [74]. Nevertheless, as exposed in this review, the use of mixed cultures introduces complexity in process control, as microbial interactions and metabolic pathways are not yet fully understood. In particular, the mechanisms governing the microbial “decision-making” between different end-products (e.g., H_2_ vs. VFAs) remain unclear, complicating efforts to steer the process toward desired outcomes.

Finally, regulatory barriers also pose a major obstacle to commercialisation. A notable example is the Steelanol project by ArcelorMittal, which converts carbon-rich gases from the steel production blast furnace into ethanol. Despite its technological success, the facility faces restrictions under European Union (EU) regulations that currently prevent the commercialisation of its ethanol product [110]. This regulatory uncertainty not only threatens the viability of the Steelanol plant but also discourages other companies from investing in syngas fermentation technologies.

In summary, while syngas fermentation holds significant potential for the sustainable production of H_2_ and VFAs, its industrial deployment is constrained by technical, biological, and regulatory challenges. Addressing these issues through targeted research and policy support will be essential to unlocking the full potential of this technology.

## 6. Future Perspectives

Syngas fermentation has been proven to be feasible at a commercial scale, as demonstrated by the application of this technology by different companies [9]. However, these industrial applications rely on pure microbial cultures, which require aseptic conditions and specialised infrastructure, factors that contribute to high capital and operational costs [74]. In this context, the use of microbial consortia emerges as a promising alternative for cost reduction, owing to their adaptability, resilience, and possibility of operating under non-sterile conditions. Additionally, microbial consortia are readily available, such as digestate from the AD process [104], which eliminates the need to purchase and maintain pure strains.

To unlock the full potential of microbial consortia in syngas fermentation, several key areas must be addressed in the future. First, a deep understanding of microbial interactions and metabolic dynamics within microbial consortia is essential to improve process yields and reproducibility. Future research should focus on systematically evaluating how different process parameters (e.g., gas composition, pH, temperature, etc) affect microbial community structure and function. Advanced genomic tools and metabolic modelling could play a crucial role in elucidating these complex interactions.

Moreover, strategies to reduce the cost of culture media should be explored to optimise the conversion in an economically feasible process. The elucidation of nutrients required for the syngas conversion into specific products, such as H_2_ and VFA, is fundamental to achieve an efficient cost reduction without impacting the yield. Additionally, including the use of industrial by-products and/or nutrient recycling could also enhance the viability of large-scale operations.

Finally, regulatory frameworks must evolve to support the commercialisation of syngas-derived products, such as hydrogen and VFAs. A comprehensive assessment of the current legislation and market pathways is needed to identify and address legal and policy barriers. Engaging stakeholders from both the public and private sectors will be vital to fostering investment, innovation, and societal acceptance of this emerging technology.

In summary, although significant advancements have been made in syngas fermentation in recent years, its future success depends on interdisciplinary efforts to optimise microbial systems, reduce costs, and ensure that regulatory frameworks align with technological advancements.

## 7. Conclusions

Based on several scientific studies, this review highlights the significant potential of manipulating the microbial “decision” toward desired products, such as H_2_ and VFA, through the strategic manipulation of process parameters, without relying on metabolic engineering or genetic modification tools. From a broad range of studies, this review has synthesised evidence showing that operational conditions are not merely control variables but powerful levers that influence microbial community dynamics and metabolic pathways, governed by both kinetic and thermodynamic principles.

From the previous studies discussed in this review, the application of the suggested specific strategies, such as maintaining moderate concentrations of H_2_ and CO_2_, optimising nitrogen and trace metal availability (particularly for hydrogenase activity), and applying short HRTs, can enhance H_2_ production by improving thermodynamic feasibility. Conversely, conditions favouring VFA production include high H_2_ partial pressures, sufficient phosphorus availability, and short HRTs to suppress methanogens and promote acidogenesis.

While these findings are grounded in the existing literature, this review contributes to integrating these insights into a coherent framework that positions process control as a cost-effective alternative to genetic interventions. Future research should focus on validating these strategies in dynamic, mixed-culture systems and exploring their scalability in continuous or industrial-scale operations. Additionally, investigating the interplay between nutrient limitations, redox balance, and microbial adaptation could further refine our understanding of how to direct microbial “decisions” toward specific metabolic routes.

## Figures and Tables

**Table 1:** Maximum CO partial pressure tolerance by different species.

Strain	Maximum CO Tolerance (mbar)	Reference
*C. aceticum*	5.4	[55]
*C. autoethanogenum*	~ 600	[54]
*C. hydrogenoformans*	~ 1000	[57]
*C. carboxidivorans*	~ 1115	[62]

**Table 2:** Summary of the pH, temperature, and end-products from syngas fermentation studies.

Inoculum	pH	Temperature (°C)	Products	Reference
Enriched anaerobic sludge	8.5	28	VFA	[79]
*Thermococcus onnurineus*	6.5	80	H_2_	[80]
Anaerobic sludge	4.5	20	Acetate	[81]
Waste-activated sludge	7.5	35	VFA	[82]
Sewage sludge	9.0	20/37	Acetate	[83]
Cow manure	7.0	37	Acetate	[84]
Domestic wastewater sludge	7.18	37	Acetate	[85]
*Clostridium carboxidivorans*	-	35	Ethanol and VFA	[62]
*Thermococcus onnurineus* NA1	6.5	80	H_2_	[86]
*Clostridium carboxidivorans* P7	6.0	-	Ethanol and acetic acid	[87]
Anaerobic sludge	6.0/9.0	65	H_2_ and VFA	[27]

**Table 3:** Trace metals and their impact on cell functions.

Trace metal	Impact on	Reference
Ni^2+^	HYD, cell growth	[69,94]
Fe^2+^	HYD	[69]
Co^2+^	CoFeS-P, ACS	[95]
Mo	Cell growth, FDH	[96]
Cu	MTFS	[97]
Zn	HYD, MTFS, FDH	[97]
Mn	HYD, MTFS	[97]

HYD: hydrogenases; CoFeS-P: corrinoid iron–sulphur protein; ACS: acetyl-CoA synthase; FDH: formate dehydrogenase; MTFS: methyltransferase.

**Table 4:** Optimum operation parameters (HRT, gas flow, and cell density) and the main end-products of the process.

Inoculum	HRT (day)	Gas Flow (vvm)	Cell Density	Main End-Product	Reference
Anaerobic sludge	2.5	-	-	VFA	[102]
Anaerobic sludge	5	-	18 g VSs/ L/ d	H_2_	[103]
Thermophilic mixed microbial consortium	8	~0.05	-	CH_4_	[104]
*C. carboxidivorans* strain P7	~2	~0.04	-	Ethanol	[105]
Anaerobic granular sludge	-	-	20–30 g VSSs/ L	H_2_	[27]
*C. ljungdahlii*	~1.4	~0.04	3.15 g/ L	Ethanol	[97]

vvm: volume of gas per volume of liquid per minute; VSs: volatile solids; VSSs: volatile suspended solids.

## Data Availability

No new data have been created.
